# Capnography sensor use is associated with reduction of adverse outcomes during gastrointestinal endoscopic procedures with sedation administration

**DOI:** 10.1186/s12871-017-0453-9

**Published:** 2017-11-28

**Authors:** Michael W. Jopling, Jiejing Qiu

**Affiliations:** 1 NorthStar Anesthesia, Springfield Regional Medical Center, Springfield, OH USA; 2 Health Economics and Outcomes Research, Medtronic, Mansfield, MA USA

**Keywords:** Gastrointestinal disorders, Endoscopic procedures, Procedural sedation, Capnography, Patient monitoring, Adverse events

## Abstract

**Background:**

Evidence to date suggests that capnography monitoring during gastrointestinal endoscopic procedures (GEP) reduces the incidence of hypoxemia, but the association of capnography monitoring with the incidence of other adverse outcomes surrounding these procedures has not been well studied. Our aims were to estimate the incidence of pharmacological rescue events and death at discharge from an inpatient or outpatient hospitalization where GEP was performed with sedation, and to determine if capnography monitoring was associated with reduced incidence of these adverse outcomes.

**Methods:**

This retrospective Premier Database analysis included medical inpatients and all outpatients undergoing GEP with sedation. Patients were grouped as follows: (1) pulse oximetry (SpO_2_) only, (2) capnography only, (3) SpO_2_ with capnography, and (4) neither SpO_2_ nor capnography. Multivariable logistic regression and propensity-score matching were used to compare patients with capnography sensor use to patients with only SpO_2_ sensor use. Outcome measures included the incidence of pharmacological rescue events, as defined by administration of naloxone and/or flumazenil, and death.

**Results:**

Two hundred fifty eight thousand and two hundred sixty two inpatients and 3,807,151 outpatients were analyzed. For inpatients, capnography monitoring was associated with a 47% estimated reduction in the odds of death at discharge (OR: 0.53 [95% CI: 0.40–0.70]; *P* < 0.0001) and a non-significant 10% estimated reduction in the odds of pharmacological rescue event at discharge (0.91 [0.65–1.3]; *P* = 0.5661). For outpatients, capnography monitoring was associated with a 61% estimated reduction in the odds of pharmacological rescue event at discharge (0.39 [0.29, 0.52]; *P* < 0.0001) and a non-significant 82% estimated reduction in the odds of death at discharge (0.18 [0.02, 1.99]; *P* = 0.16).

**Conclusions:**

In hospital medical inpatients and all outpatients undergoing GEP performed with sedation, capnography monitoring was associated with a reduced likelihood of pharmacological rescue events in outpatients and death in inpatients when assessed at discharge. Despite the limitations of the retrospective data analysis methodology, the use of capnography during these procedures is recommended.

**Electronic supplementary material:**

The online version of this article (10.1186/s12871-017-0453-9) contains supplementary material, which is available to authorized users.

## Background

Gastrointestinal endoscopic procedures (GEP) such as esophagogastroduodenoscopy (EGD), endoscopic retrograde cholangiopancreatography (ERCP), and colonoscopy are standard procedures for the diagnosis and therapy of gastrointestinal disorders, but can be associated with patient discomfort. To improve patient comfort, the use of sedation in these procedures is common. However, the use of sedative agents can result in drug-induced airway obstruction, respiratory depression with hypoventilation, and hypoxemia [1–6]. Cardiopulmonary adverse events remain a leading cause of morbidity and mortality during GEP with procedural sedation [3–6].

Given these concerns and potential sequelae, patient monitoring guidelines for procedural sedation have typically recommended continuous pulse oximetry combined with visual assessment of a patient’s breathing pattern [7, 8]. Despite the value of these assessments, monitoring of arterial oxygen saturation (SaO_2_) via pulse oximetry does not necessarily provide a satisfactory assessment of the adequacy of ventilation. Importantly, significant alveolar hypoventilation can occur in the presence of normal SaO_2_ as shown by pulse oximetry (SpO_2_) and inadequate ventilation can precede hypoxemia by several minutes [1, 9, 10]. The risks of procedural sedation are further compounded by the fact that GEP with sedation administration are often conducted without an anesthesia provider present and the delivery of care is performed in a remote hospital location rather than within the primary operating room suite [11].

Given these potential limitations of pulse oximetry and the recognized need for improved patient monitoring during GEP with sedation administration, the use of capnography to monitor end-tidal carbon dioxide (ETCO_2_) is increasingly common [1, 12–14]. Sidestream and mainstream capnography, by continuously monitoring ETCO_2_ levels, respiratory rate, and waveform pattern, allow for the near real-time assessment of ventilation in spontaneously breathing patients and provide a more complete assessment of the adequacy of ventilation than either SpO_2_ or visual inspection of breathing [9, 15–17]. As such, capnography has been clinically demonstrated to provide an earlier indicator of respiratory distress than SpO_2_ alone [9, 13]. Several studies have shown that the addition of capnography monitoring during GEP with procedural sedation results in a significant reduction in the incidence of hypoxemia [1, 12–14].

While published evidence to date suggests that capnography monitoring during GEP reduces the incidence of hypoxemia, the association of capnography monitoring with incidence of adverse outcomes when these procedures are performed with sedation has been insufficiently studied. Thus, the aims of this analysis were to estimate the incidence of pharmacological rescue events and death as assessed upon discharge from an inpatient or outpatient hospitalization during which GEP was performed with sedation, separately, for matched patients with and without capnography monitoring using an administrative database.

## Methods

### Patient population

This was a retrospective analysis of patient data (January 2008 to December 2013) from the Premier Healthcare database (Premier Inc., Charlotte, North Carolina), a privately owned administrative database that is one of the largest hospital-level resource utilization and economic databases in the United States, representing approximately 13% of inpatient hospitalizations annually [18]. Discharge-level data includes information on patient and provider characteristics, International Classification of Diseases 9th revision Clinical Modification (ICD-9-CM) diagnosis and procedure codes, hospital resource utilization such as specific device usage, medications and laboratory services, discharge disposition (including death), and charges/cost data on all entries. Outcomes for this study were selected based on sedation-related adverse events that could be discretely identified within this retrospective administrative database, and included administration of reversal agents naloxone and/or flumazenil administered on the day of the GEP procedure, and mortality.

Mortality was assessed by identification of patients who were deceased upon hospital discharge. Due to the nature of the retrospective administrative database, causality of mortality cannot be inferred; rather, our intent was to describe the association between GEP under sedation and death at discharge in propensity-matched patient populations.

Data within the Premier database are de-identified in accordance with the Health Insurance Portability and Accountability Act (HIPAA). Per United States Title 45 CFR (Code of Federal Regulations) Part 46.101, institutional review board (IRB) approval for this study was not required under the exemption that this research involved the study of existing data and that the information was recorded in such a manner that the subjects could not be identified, directly or through identifiers linked to the subjects.

**Fig. 1:**
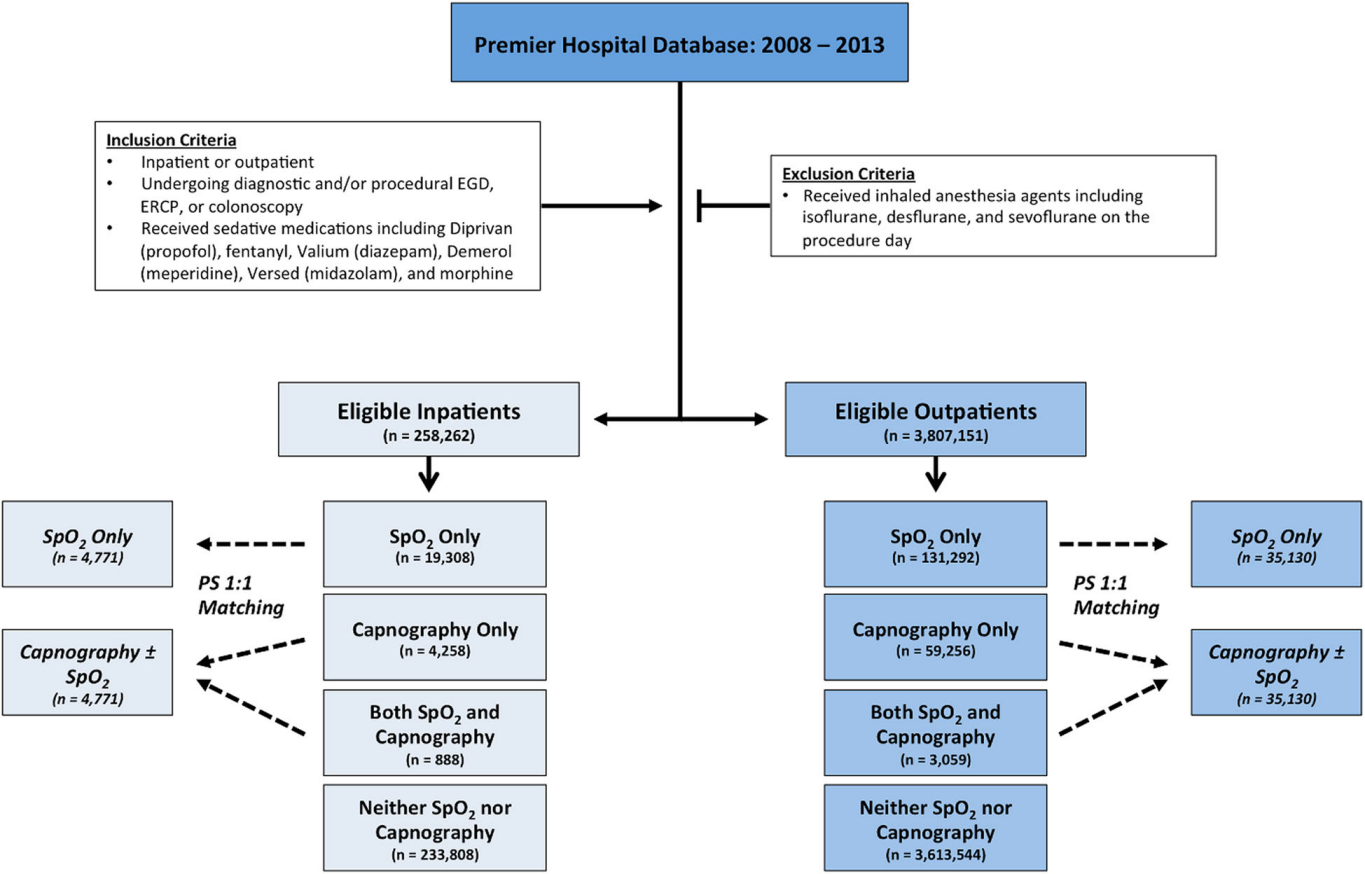
Study Design and Patient Disposition. The Premier Hospital Database from 2008 to 2013 was queried for eligible patients who underwent selected gastrointestinal endoscopic procedures with procedural sedation. Eligible inpatients and outpatients were grouped into four groups: (1) SpO_2_ only, (2) capnography only, (3) both SpO_2_ and capnography, and (4) neither SpO_2_ nor capnography. Eligible patients were matched 1:1 using propensity score matching techniques to generate the final analysis groups of (1) SpO_2_ only and (2) capnography (± SpO_2_). EGD = esophagogastroduodenoscopy; ERCP = endoscopic retrograde cholangiopancreatography

Patients were included in the analysis if they were medical inpatients or any outpatients undergoing diagnostic and procedural EGD, ERCP, or colonoscopy as identified using a combination of CPT/ICD-9 codes (see Additional file 1: Table S1). Patients with documentation of sedative medications (propofol, fentanyl, diazepam, meperidine, midazolam, and morphine) were included, while patients who received an inhaled anesthesia agent (isoflurane, desflurane, and sevoflurane) on the procedure day were excluded (Fig. 1). Patients who were monitored with capnography and oximetry were identified using medical and surgical supply or respiratory supply billing data indicating that a capnography and/or oximetry sensor was used on and 1-day post-procedure (see Additional file 2: Table S2 for a list of capnography sensor supply codes). For description of the overall population, patients were grouped into four mutually exclusive categories: (1) SpO_2_ only, (2) capnography only, (3) SpO_2_ with capnography, and (4) neither SpO_2_ nor capnography. For the statistical analyses described below, patients were grouped into two mutually exclusive categories: capnography (with and without SpO_2_) and SpO_2_ only (see Fig. 1).

### Statistical analysis

Descriptive statistics including frequencies and proportions were used for categorical data; means and standard deviations (SD) for continuous variables were calculated for patient demographics and hospital characteristics. Due to the differences between patients with capnography sensor use and patients who had SpO_2_ sensor use only, the analysis was conducted using Propensity Score (PS) methodology to match patients in order to reduce bias. This matching was done separately for the inpatient and outpatient populations. For the PS matching, a greedy algorithm using the nearest available pair matching method was conducted to match patients on capnography monitoring (± SpO_2_ monitoring) to those with SpO_2_ sensor only on a 1 to 1 ratio [19]. The propensity scores were estimated using a logistic regression model adjusted for patient information such as age, gender, race, comorbidities defined by the Charlson Comorbidity Index (CCI) including myocardial infarction, congestive heart failure, dementia, chronic obstructive pulmonary disease (COPD), rheumatoid arthritis, peptic ulcer disease, paralysis, chronic renal failure, cancer, metastatic solid tumor, AIDS, obesity, diabetes, hypertension, peripheral vascular disease, cardiovascular disease, mild liver disease and moderate-severe liver disease, admission type (elective or emergency), and hospital characteristics such as region, bed size, rural vs. urban, and teaching vs. non-teaching (see Additional file 3: Table S3). Due to the large sample size in this analysis (35,130 matched outpatient pairs and 4771 matched inpatient pairs), the standardized differences were calculated and compared to a cutoff of 0.1 in order to measure the balance of the matched groups, as this balance measure is not influenced by sample size [20].

After matching, the associations between capnography monitoring and outcomes of interest were estimated using multivariable logistic regression analysis adjusted for all the variables noted above for the PS methodology (see Additional file 3: Table S3), and the association estimates were supported by chi-square tests. These analyses were conducted for the inpatient and outpatient populations separately. The outcomes of interest were pharmacologic rescue events, as defined by administration of naloxone and/or flumazenil, and death, between patients with capnography monitoring (± SpO_2_ monitoring) and patients with SpO_2_ sensor use only, for inpatients and outpatients, separately. The significance level was considered to be 0.05. Due to the multiple outcomes included in the study, the Bonferroni method was employed to adjust the significance level, therefore a *P*-value less than 0.025 was considered as statistically significant. All statistical analyses were conducted using SAS® 9.2 for UNIX (Cary, NC, USA).

## Results

### Incidence and patient demographics

Overall, the analysis identified 258,262 eligible inpatients and 3,807,151 eligible outpatients (Fig. 1 and Table 1). For the inpatient population, 19,308 (7.5%) patients received SpO_2_ only, 4258 (1.6%) received capnography only, 888 (0.3%) received both capnography and SpO_2_, and 233,808 (90.5%) received neither capnography nor SpO_2_. For the outpatient population, 131,292 (3.4%) received SpO_2_ only, 59,256 (1.6%) received capnography only, 3059 (0.1%) received both capnography and SpO_2_, and 3,613,544 (94.9%) received neither capnography nor SpO_2_ (Table 1 and Fig. 1).

**Table 1:** Demographics – overall population

Characteristic	Inpatient (*n* = 258,262)				Outpatient (*n* = 3,807,151)			
	SpO_2_ Only (*n* = 19,308)	Capnography Only (*n* = 4258)	SpO_2_ and Capnography (*n* = 888)	Neither (*n* = 233,808)	SpO_2_ Only (*n* = 131,292)	Capnography Only (*n* = 59,256)	SpO_2_ and Capnography (*n* = 3059)	Neither (*n* = 3,613,544)
Age								
Overall	63.43 ± 18.42	64.12 ± 17.19	65.02 ± 17.05	64.33 ± 17.52	53.36 ± 20.91	58.35 ± 14.70	57.95 ± 15.24	57.54 ± 15.31
80+	3961 (21%)	854 (20%)	204 (23%)	50,707 (22%)	8260 (6%)	3249 (5%)	183 (6%)	200,883 (6%)
71–80	3886 (20%)	911 (21%)	168 (19%)	47,930 (21%)	18,818 (14%)	9147 (15%)	458 (15%)	515,643 (14%)
61–70	3733 (19%)	817 (19%)	181 (20%)	43,336 (19%)	27,376 (21%)	14,813 (25%)	729 (24%)	857,286 (24%)
51–60	3399 (18%)	731 (17%)	158 (18%)	40,155 (17%)	30,363 (23%)	16,354 (28%)	792 (26%)	1,038,536 (28%)
41–50	2193 (11%)	517 (12%)	105 (12%)	27,244 (12%)	17,515 (13%)	8913 (15%)	520 (17%)	55,431 (15%)
31–40	1039 (5%)	237 (6%)	48 (5%)	14,190 (6%)	8924 (7%)	3852 (7%)	223 (7%)	233,897 (6%)
18–30	752 (4%)	183 (4%)	17 (2%)	9253 (4%)	6412 (5%)	2597 (4%)	119 (4%)	158,524 (4%)
< 18	345 (2%)	8 (0%)	7 (1%)	993 (0%)	13,624 (10%)	331 (1%)	35 (1%)	53,344 (1%)
CCI								
Overall	2.59 ± 2.49	2.53 ± 2.48	2.57 ± 2.47	2.52 ± 2.46	0.51 ± 1.04	0.45 ± 0.97	0.79 ± 1.20	0.39 ± 0.90
> 2	8163 (42%)	1743 (41%)	368 (41%)	95,025 (41%)	6283 (5%)	2310 (4%)	228 (7%)	119,647 (3%)
2	2990 (15%)	637 (15%)	148 (17%)	36,636 (16%)	8371 (6%)	3302 (6%)	331 (11%)	171,185 (5%)
1	3722 (19%)	894 (21%)	169 (19%)	49,112 (21%)	24,123 (18%)	10,705 (18%)	824 (27%)	571,858 (16%)
0	4433 (23%)	984 (23%)	203 (23%)	53,035 (23%)	92,515 (70%)	42,939 (72%)	1676 (55%)	2,750,854 (76%)
APR severity of illness								
1 = mild	1751 (9%)	443 (10%)	87 (10%)	23,466 (10%)	0 (0%)	0 (0%)	0 (0%)	0 (0%)
2 = moderate	5621 (29%)	1428 (34%)	304 (34%)	75,251 (32%)	0 (0%)	0 (0%)	0 (0%)	0 (0%)
3 = severe	8283 (43%)	1816 (43%)	360 (41%)	98,750 (42%)	0 (0%)	0 (0%)	0 (0%)	0 (0%)
4 = extreme	3653 (19%)	571 (13%)	137 (15%)	36,341 (16%)	0 (0%)	0 (0%)	0 (0%)	0 (0%)
Gender								
Female	9975 (52%)	2224 (52%)	450 (51%)	124,726 (53%)	74,730 (57%)	33,635 (57%)	1867 (61%)	2,024,108 (56%)
Male	9333 (48%)	2034 (48%)	438 (49%)	109,082 (47%)	56,562 (43%)	25,621 (43%)	1192 (39%)	1,589,436 (44%)
Race								
White	14,032 (73%)	2837 (67%)	580 (65%)	158,011 (68%)	101,037 (77%)	45,307 (76%)	2689 (88%)	2,637,723 (73%)
Black	2756 (14%)	731 (17%)	177 (20%)	34,632 (15%)	11,813 (9%)	6380 (11%)	284 (9%)	304,535 (8%)
Hispanic	385 (2%)	77 (2%)	87 (10%)	7084 (3%)	3987 (3%)	1234 (2%)	0 (0%)	66,752 (2%)
Other	2135 (11%)	613 (14%)	44 (5%)	34,081 (15%)	14,455 (11%)	6335 (11%)	86 (3%)	604,534 (17%)
Comorbidity								
HTN	12,079 (63%)	2747 (65%)	582 (66%)	144,259 (62%)	40,244 (31%)	18,788 (32%)	1451 (47%)	972,692 (27%)
Diabetes	5930 (31%)	1354 (32%)	274 (31%)	71,495 (31%)	17,501 (13%)	7797 (13%)	615 (20%)	393,796 (11%)
COPD	4727 (25%)	1031 (24%)	209 (24%)	55,507 (24%)	11,656 (9%)	4524 (8%)	570 (19%)	254,671 (7%)
CRF	4070 (21%)	873 (21%)	183 (21%)	46,587 (20%)	1658 (1%)	593 (1%)	63 (2%)	35,807 (1%)
CHF	3688 (19%)	681 (16%)	148 (17%)	41,238 (18%)	2281 (2%)	665 (1%)	113 (4%)	33,729 (1%)
PUD	2748 (14%)	577 (14%)	98 (11%)	33,639 (14%)	5453 (4%)	2045 (4%)	162 (5%)	111,461 (3%)
Obesity	2125 (11%)	461 (11%)	100 (11%)	25,189 (11%)	6027 (5%)	1644 (3%)	143 (5%)	130,982 (4%)
Cancer	1895 (10%)	449 (11%)	86 (10%)	23,155 (10%)	3970 (3%)	1611 (3%)	85 (3%)	85,219 (2%)
MLD	2011 (10%)	417 (10%)	98 (11%)	23,108 (10%)	1913 (2%)	808 (1%)	63 (2%)	39,733 (1%)
MSLD	2009 (10%)	399 (9%)	91 (10%)	22,687 (10%)	2193 (2%)	917 (2%)	66 (2%)	43,521 (1%)
MI	1713 (9%)	348 (8%)	72 (8%)	21,179 (9%)	2171 (2%)	982 (2%)	115 (4%)	49,888 (1%)
CVD	1303 (7%)	256 (6%)	54 (6%)	15,601 (7%)	764 (1%)	183 (<1%)	36 (1%)	11,995 (<1%)
PVD	1406 (7%)	281 (7%)	63 (7%)	15,224 (7%)	1081 (1%)	340 (1%)	57 (2%)	17,958 (<1%)
MST	916 (5%)	218 (5%)	50 (6%)	11,248 (5%)	496 (<1%)	190 (<1%)	13 (<1%)	9539 (<1%)
RA	657 (3%)	144 (3%)	16 (2%)	8177 (4%)	1453 (1%)	693 (1%)	60 (2%)	29,597 (1%)
Dementia	172 (1%)	24 (1%)	12 (1%)	2127 (1%)	39 (<1%)	19 (<1%)	2 (<1%)	733 (<1%)
Paralysis	172 (1%)	29 (1%)	7 (1%)	2334 (1%)	91 (<1%)	23 (<1%)	6 (<1%)	1459 (<1%)
AIDS	65 (<1%)	15 (<1%)	10 (1%)	978 (<1%)	72 (<1%)	27 (<1%)	2 (<1%)	1687 (<1%)
Region								
E. N. Central	707 (4%)	864 (20%)	19 (2%)	36,866 (16%)	900 (1%)	7311 (12%)	30 (1%)	747,309 (21%)
E. S. Central	2175 (11%)	7 (<1%)	41 (5%)	12,738 (5%)	22,514 (17%)	113 (<1%)	1886 (62%)	218,449 (6%)
M. Atlantic	1097 (6%)	12 (<1%)	0 (0%)	23,470 (10%)	148 (<1%)	8 (<1%)	0 (0%)	195,389 (5%)
Mountain	434 (2%)	370 (9%)	0 (0%)	11,684 (5%)	1029 (1%)	2959 (5%)	2 (<1%)	205,205 (6%)
New England	446 (2%)	108 (3%)	0 (0%)	8113 (3%)	2281 (2%)	7431 (13%)	1 (<1%)	193,418 (5%)
Pacific	1584 (8%)	380 (9%)	23 (3%)	34,221 (15%)	11,604 (9%)	3170 (5%)	69 (2%)	572,954 (16%)
S. Atlantic	8637 (45%)	2122 (50%)	745 (84%)	67,627 (29%)	53,391 (41%)	31,137 (53%)	776 (25%)	884,648 (24%)
W. N. Central	603 (3%)	80 (2%)	8 (1%)	16,276 (7%)	8190 (6%)	355 (1%)	7 (<1%)	370,834 (10%)
W. S. Central	3625 (19%)	315 (7%)	52 (6%)	22,813 (10%)	31,235 (24%)	6772 (11%)	288 (9%)	225,338 (6%)
Teaching Hospital								
Yes	8672 (45%)	804 (19%)	130 (15%)	88,195 (38%)	41,105 (31%)	9659 (16%)	491 (16%)	1,117,242 (31%)
Hospital Bed size								
< 250	2523 (13%)	822 (19%)	81 (9%)	52,543 (22%)	38,376 (29%)	17,738 (30%)	1968 (64%)	1303,833 (36%)
250–500	7752 (40%)	2571 (60%)	257 (29%)	112,743 (48%)	25,602 (20%)	33,685 (57%)	1022 (33%)	1,672,591 (46%)
500 +	9033 (47%)	865 (20%)	550 (62%)	68,522 (29%)	67,314 (51%)	7833 (13%)	69 (2%)	637,120 (18%)
Hospital Location								
Rural	2320 (12%)	1124 (26%)	47 (5%)	26,096 (11%)	27,118 (21%)	8916 (15%)	44 (1%)	598,635 (17%)
Urban	16,988 (88%)	3134 (74%)	841 (95%)	207,712 (89%)	104,174 (79%)	50,340 (85%)	3015 (99%)	3,014,909 (83%)

As expected, the inpatient population tended to be older than the outpatient population (mean age: 64.3 years vs. 57.4 years) with a higher mean CCI (2.53 vs. 0.39). Patients were predominantly white and approximately 50% male; the most common comorbidities included hypertension, diabetes, and COPD. Most patients were admitted to an urban hospital with ≥ 250 beds (Table 1).

### Propensity score matching

Before matching, the standardized difference scores indicated significant differences (> 0.1) in selected patient demographics for both the inpatient and outpatient populations (Additional files 4, and 5: Tables S4 and S5). After matching, there were 4771 matched inpatients and 35,130 matched outpatients who received capnography monitoring (± SpO_2_ monitoring) or SpO_2_ monitoring only, and most differences in patient demographics were accounted for by the matching process.

### Capnography and the risk of adverse events

**Fig. 2:**
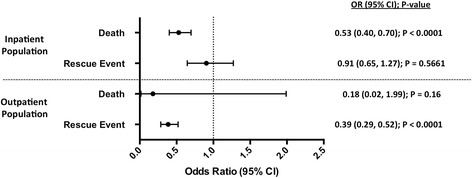
Propensity Score Matched Odds Ratios. Adjusted Odds Ratios (95% CI) for death and rescue event (naloxone and/or flumazenil administration) for capnography (± SpO_2_) as compared to SpO_2_ only, for the matched inpatient and outpatient populations. CI = confidence interval; OR = odds ratio

Patient outcomes and multivariable logistic regression analysis for the PS matched patients are presented in Fig. 2 and Additional files 6 and 7: Tables S6 and S7. For the PS matched inpatient population, there were 94 (1.97%) deaths and 66 (1.38%) pharmacologic rescue events in patients receiving capnography (± SpO_2_ monitoring) as compared to 166 (3.48%) deaths and 74 (1.55%) pharmacologic rescue events in patients receiving SpO_2_ monitoring only (Additional file 6: Table S6). For the PS matched outpatient population, there was 1 (< 0.01%) death and 63 (0.18%) pharmacologic rescue events in patients receiving capnography (± SpO_2_ monitoring) as compared to 4 (0.01%) deaths and 148 (0.42%) pharmacologic rescue events in patients receiving SpO_2_ monitoring only (Additional file 7: Table S7).

Overall, the use of capnography was associated with a 47% estimated reduction in the odds of death (OR: 0.53; 95% CI: 0.40, 0.70; *P* < 0.0001) for the inpatient population and a 61% estimated reduction in the odds of pharmacological rescue event (OR: 0.39; 95% CI: 0.29, 0.52; *P* < 0.0001) for the outpatient population (Fig. 2). Capnography was also associated with non-significant estimated reductions in the odds of pharmacological rescue event (OR: 0.91; 95% CI: 0.65, 1.27; *P* = 0.5661) for the inpatient population and the odds of death (OR: 0.18; 95% CI: 0.02, 1.99; *P* = 0.16) for the outpatient population (Fig. 2).

Chi-Square analysis of the PS matched samples revealed similar odds ratios as the multivariable logistic regression analysis. More specifically, for the inpatient population, the use of capnography monitoring was associated with a significant estimated reduction in the odds of death (OR: 0.56; 95% CI: 0.43, 0.72, *P* < 0.0001) while for the outpatient population, the use of capnography monitoring was associated with a significant estimated reduction in the odds of rescue medication use (OR: 0.42; 95% CI: 0.32, 0.57; *P* < 0.0001) (Table 2).

**Table 2:** Chi-Square using PS Matched Samples

Patient population	Outcome		Capnography		*P*-value	OR
			Yes	No		
Inpatient	Death	Yes	94 (2.0%)	166 (3.5%)	< 0.0001	0.56 (0.43, 0.72)
		No	4,677 (98.0%)	4,605 (96.5%)		
	Rescue Event	Yes	66 (1.4%)	74 (1.6%)	0.50	0.89 (0.64, 1.24)
		No	4,705 (98.6%)	4,697 (98.5%)		
Outpatient	Death	Yes	ND	ND	ND	ND
		No				
	Rescue Event	Yes	63 (0.2%)	148 (0.4%)	< 0.0001	0.42 (0.32, 0.57)
		No	35,067 (99.8%)	34,982 (99.6%)		

*ND* not determined, *OR* odds ratio

## Discussion

The results of this large database analysis indicate that the use of capnography was associated with a 47% estimated reduction in the odds of death for the matched inpatient population and a 61% estimated reduction in the odds of pharmacological rescue event for the matched outpatient population. For the matched inpatient population, the use of capnography was associated with a reduction in both mortality and pharmacologic rescue rates, though reduction in pharmacologic rescue rates was not statistically significant. The reason for close rates of pharmacologic rescue in the inpatient setting may be explained by several factors. First, patients with more monitors may be more closely watched by attending clinicians, who may check both alarms and patients. Second, in the inpatient setting, resuscitation may proceed immediately to mask bag ventilation or endotracheal intubation instead of reliance on pharmacologic reversal agents, whereas in the outpatient setting where anesthesia personnel may be limited, pharmacologic reversal may be preferred to primary airway control. Lastly, the fact that the outpatient group demonstrated a reduction in pharmacologic rescue but not death rates may be related to patient flow in the outpatient setting. If a patient’s condition declines significantly, they are likely to be admitted to hospital well before death occurs; thus, it is not surprising that mortality is rarely seen in the outpatient setting. However, inpatient admissions from the outpatient setting are not reliably retrievable from this retrospective database, so this analysis is not presented here. Regardless, to our knowledge, these data provide the first evidence that capnography use during GEP with procedural sedation is associated with a reduction in the odds of these adverse outcomes.

Procedural sedation can result in loss of protective pharyngeal airway reflexes, upper airway obstruction, central respiratory depression, alveolar hypoventilation, atelectasis, hypercapnia and hypoxemia [21]. Cardiopulmonary adverse events remain a leading cause of morbidity and mortality with GEP [3–6]. In a study of 21,011 procedures using midazolam and/or diazepam, Arrowsmith et al. reported serious cardiopulmonary complications in 5.4 per 1000 procedures [4]. Similarly, in a database analysis of over 300,000 endoscopic procedures with sedation, Sharma et al. reported an incidence of 9.3 cardiopulmonary adverse events per 1000 procedures [6]. These authors also noted that the presence of the endoscope across the upper airway along with a depressed respiratory drive due to the sedative medications in combination with the inability to accurately assess ventilation using pulse oximetry can result in undetected hypoventilation and a higher incidence of cardiopulmonary adverse events [6]. Importantly, the risks of GEP adverse events exceed those typically reported for general anesthesia procedures performed outside the operating room [11].

Given these risks of procedural sedation and the fact that sedation levels during GEP often approach those of general anesthesia, [2] adequate patient monitoring is critical, especially with these types of procedures often conducted by non-anesthesiologists outside of the operating room, where personnel and equipment availability can limit effective response to acute deterioration. In fact, in an analysis of 63,000 patients undergoing diagnostic or therapeutic procedures under sedation or anesthesia, over 40% of patients were sedated by non-anesthesia providers and 12.4% of the anesthesiology cases were performed outside of the operating room [22].

Historically, patient monitoring during GEP has focused on pulse oximetry in combination with vital sign monitoring and visual inspection of ventilation [7, 8]. Unfortunately, as noted above, monitoring arterial oxygen saturation via pulse oximetry, especially in patients receiving supplemental oxygen therapy, is inadequate for effectively detecting the onset of hypoventilation [1, 9, 10, 23]. Importantly, several closed claims analyses have indicated that the application of better monitoring, including capnography, could have prevented nearly half of claims associated with oversedation [11, 24]. To date, a number of studies have indicated that the addition of capnography to standard monitoring provides superior detection of respiratory depression during procedural sedation [10, 12, 13, 25–27]. These data and others have led to the recent recognition of capnography as a critical component of adequate patient monitoring during moderate or deep sedation by the American Society of Anesthesiologists (ASA) [28]. In addition, several recent cost-benefit analyses support the use of capnography [29, 30]. Despite the apparent clinical and economic benefits of capnography and support by the ASA and other governing bodies, the use of capnography in these types of procedures remains relatively low.

### Limitations

We note that this retrospective database analysis has several limitations. While we are able to demonstrate an association between capnography use and a reduction in adverse outcomes, we cannot demonstrate causation. Additionally, we are unable to characterize the training, education, experience and expertise of those monitoring, interpreting and acting upon the results of monitoring. Finally, with an administrative database, we are unable to characterize adverse events that cannot be described by discharge fields on the hospital chargemaster; limiting adverse events by pharmacologic reversal agents and mortality certainly underestimates to total number of adverse events but appropriately characterizes those serious adverse events that may have longstanding patient or hospital implications. However, for questions such as these, the ability to conduct a well-controlled, randomized study of such a size to provide statistically relevant results is limited by ethical considerations and standard of care guidelines that now recommend the use of capnography in these types of procedures. The cost of performing such a study is also likely to be prohibitive. Despite the inherent limitations of a database analysis, we believe that our results are strengthened by the large sample size and the use of propensity score matching to generate well-matched patient populations.

Another concern with a retrospective database analysis lies in the use CPT/ICD-9 codes to accurately identify patients of interest and to effectively capture the use of specific patient monitoring equipment. It is important to note that our analysis only reports events observed in the two patient populations (capnography ± SpO_2_ vs. SpO_2_ only) for whom patient monitoring was reported and that we did not infer any conclusions from the patient population for which no monitoring was reported. We recognize that the relatively large group of patients for whom neither SpO_2_ nor capnography monitoring were reported may be improbable, but given our reliance on billing codes to identify the monitoring equipment utilized, cases involving reusable sensors that were not included in the billing record would have been omitted from the analysis. Given the availability of reusable SpO_2_ sensors, it is likely that our analysis underestimates the use of SpO_2_ monitoring. It is in part due to this limitation that we grouped capnography only patients with capnography plus SpO_2_ patients in our final analysis, in that we find it unlikely that a patient would receive capnography monitoring without SpO_2_ monitoring. While there are known methods of CO_2_ sampling that do not require a specific sensor, the use of these technologies was more common prior to the availability of capnography-specific patient interface sensors (which do not utilize reusable sensors), thus errors of omission with respect to capnography monitoring are less likely.

Despite these limitations regarding sensor use, we believe that the combination of procedural and diagnostic codes employed was optimized for the current analysis. Also, due to the discharge-level nature of the data contained in the Premier database, we cannot directly comment on the specific timing of patient monitoring and/or medication administration relative to the GEP of interest. An additional limitation of the Premier database is that certain variables, including patient BMI and history of comorbidities such as sleep apnea, are not captured in the database and thus are not available for inclusion in the propensity score calculations, which might potentially impact the estimation of the capnography effect.

### Implications of findings

The addition of capnography to procedural monitoring without understanding how to optimize the information it provides is unlikely to be very useful. The Centers for Medicare and Medicaid Services (CMS) *Conditions of Participation* requires that all anesthesia services (whether administered by an anesthesia-trained provider or not) in a hospital facility be organized under the directorship of an anesthesiologist. This study may help provide the necessary information to encourage appropriate monitoring standards in gastrointestinal (GI) procedure suites and to encourage and assist in providing the necessary education to utilize data from the patient monitoring devices to more effectively manage patients receiving procedural sedation. In our era of increased public awareness of patient safety events, we must guard against a normalization of deviance when dealing with relatively rare but clinically significant events. To provide safe care and minimize the potential for preventable harm, we must continue to learn from the mistakes of others to help ensure the best reasonable outcome from these extremely common procedures.

The addition of electronic monitoring or physician work to any procedure may be perceived as cost-additive. One recent study reported that the addition of capnography to an endoscopy procedural sedation monitoring protocol resulted in a 27.2% and 18.0% reduction in the proportion of patients experiencing an adverse event during deep and moderate procedural sedation/analgesia, respectively [29]. For this analysis, the authors reported that the median number needed to treat to avoid any adverse event was 8 patients for deep sedation and 6 patients for moderate sedation, and estimated that the addition of capnography reduced the cost per procedure by $85 (during deep sedation) and $35 (during moderate sedation). The authors concluded that capnography is estimated to be cost-effective if not cost-saving during procedural sedation for gastrointestinal endoscopy and suggested that the addition of capnography monitoring to the standard of care during procedural sedation for endoscopy should be considered.

## Conclusions

The results of our analysis indicate that the use of capnography during GEP with procedural sedation is associated with significant reductions in the risk of pharmacological rescue events in outpatients and death in inpatients. Despite the limitations of this retrospective data-based study, we believe the use of capnography during GEP performed with sedation should be recommended.

## Additional files

Additional file 1: Table S1. Current Procedural Terminology Codes for Esophagoscopy, Small Bowel Endoscopy, Colonoscopy, Sigmoidoscopy and Anoscopy Procedures. (DOCX 188 kb)

Additional file 2: Table S2. Capnography Sensor Codes. (DOCX 178 kb)

Additional file 3: Table S3. Variables Included in the Multivariable Logistic Regression. (DOCX 67 kb)

Additional file 4: Table S4. Propensity Score Matching – Inpatient Population. (DOCX 170 kb)

Additional file 5: Table S5. Propensity Score Matching – Outpatient Population. (DOCX 155 kb)

Additional file 6: Table S6. Patient Outcomes Before and After Propensity Score Matching – Inpatient Population. (DOCX 63 kb)

Additional file 7: Table S7. Patient Outcomes Before and After Propensity Score Matching – Outpatient Population. (DOCX 63 kb)

## Abbreviations

ASA: American Society of Anesthesiologists
CCI: Charlson Comorbidity Index
CFR: Code of Federal Regulations
CI: Confidence interval
CMS: Centers for Medicare and Medicaid Services
COPD: Chronic obstructive pulmonary disease
CPT: Current Procedural Terminology
EGD: Esophagogastroduodenoscopy
ERCP: Endoscopic retrograde cholangiopancreatography
ETCO_2_: End-tidal carbon dioxide
GEP: Gastrointestinal endoscopic procedures
GI: Gastrointestinal
HIPAA: Health Insurance Portability and Accountability Act
ICD-9-CM: International Classification of Diseases 9th revision Clinical Modification
IRB: Institutional review board
OR: Odds ratio
PS: Propensity Score
SaO_2_: Arterial oxygen saturation
SD: Standard deviation
SpO_2_: Pulse oximetry
