# Supplementary material for: Capnography sensor use is associated with reduction of adverse outcomes during gastrointestinal endoscopic procedures with sedation administration
Source: BMC Anesthesiol. 2017 Nov 28;17:157. doi: 10.1186/s12871-017-0453-9 (PMC5704394; doi:10.1186/s12871-017-0453-9)
Supplement: Supplementary file 1 — Current Procedural Terminology Codes for Esophagoscopy, Small Bowel Endoscopy, Colonoscopy, Sigmoidoscopy and Anoscopy Procedures. (DOCX 188 kb) [file 12871_2017_453_MOESM1_ESM.docx]

**Supplemental Table 1. CPT Codes for Esophagoscopy, Small Bowel Endoscopy, Colonoscopy, Sigmoidoscopy and Anoscopy Procedures**

| **Code** | **Description** |
| --- | --- |
| 43260 | Endoscopic retrograde cholangiopancreatography (ercp); diagnostic, including collection of specimen(s) by brushing or washing, when performed (separate procedure) |
| 43261 | Endoscopic retrograde cholangiopancreatography (ercp); with biopsy, single or multiple |
| 43262 | Endoscopic retrograde cholangiopancreatography (ercp); with sphincterotomy/papillotomy |
| 43263 | Endoscopic retrograde cholangiopancreatography (ercp); with pressure measurement of sphincter of oddi |
| 43264 | Endoscopic retrograde cholangiopancreatography (ercp); with removal of calculi/debris from biliary/pancreatic duct(s) |
| 43265 | Endoscopic retrograde cholangiopancreatography (ercp); with destruction of calculi, any method (eg, mechanical, electrohydraulic, lithotripsy) |
| 43275 | Endoscopic retrograde cholangiopancreatography (ercp); with removal of foreign body(s) or stent(s) from biliary/pancreatic duct(s) |
| 43276 | Endoscopic retrograde cholangiopancreatography (ercp); with removal and exchange of stent(s), biliary or pancreatic duct, including pre- and post-dilation and guide wire passage, when performed, including sphincterotomy, when performed, each stent exchanged |
| 43277 | Endoscopic retrograde cholangiopancreatography (ercp); with trans-endoscopic balloon dilation of biliary/pancreatic duct(s) or of ampulla (sphincteroplasty), including sphincterotomy, when performed, each duct |
| 43278 | Endoscopic retrograde cholangiopancreatography (ercp); with ablation of tumor(s), polyp(s), or other lesion(s), including pre- and post-dilation and guide wire passage, when performed |
| 43200 | Esophagoscopy, flexible, transoral; diagnostic, including collection of specimen(s) by brushing or washing, when performed (separate procedure) |
| 43201 | Esophagoscopy, flexible, transoral; with directed submucosal injection(s), any substance |
| 43202 | Esophagoscopy, flexible, transoral; with biopsy, single or multiple |
| 43204 | Esophagoscopy, flexible, transoral; with injection sclerosis of esophageal varices |
| 43205 | Esophagoscopy, flexible, transoral; with band ligation of esophageal varices |
| 43215 | Esophagoscopy, flexible, transoral; with removal of foreign body |
| 43216 | Esophagoscopy, flexible, transoral; with removal of tumor(s), polyp(s), or other lesion(s) by hot biopsy forceps or bipolar cautery |
| 43217 | Esophagoscopy, flexible, transoral; with removal of tumor(s), polyp(s), or other lesion(s) by snare technique |
| 43220 | Esophagoscopy, flexible, transoral; with transendoscopic balloon dilation (less than 30 mm diameter) |
| 43226 | Esophagoscopy, flexible, transoral; with insertion of guide wire followed by passage of dilator(s) over guide wire |
| 43227 | Esophagoscopy, flexible, transoral; with control of bleeding, any method |
| 43231 | Esophagoscopy, flexible, transoral; with endoscopic ultrasound examination |
| 43232 | Esophagoscopy, flexible, transoral; with transendoscopic ultrasound-guided intramural or transmural fine needle aspiration/biopsy(s) |
| 43235 | Esophagogastroduodenoscopy, flexible, transoral; diagnostic, including collection of specimen(s) by brushing or washing, when performed (separate procedure) |
| 43236 | Esophagogastroduodenoscopy, flexible, transoral; with directed submucosal injection(s), any substance |
| 43237 | Esophagogastroduodenoscopy, flexible, transoral; with endoscopic ultrasound examination limited to the esophagus, stomach or duodenum, and adjacent structures |
| 43238 | Esophagogastroduodenoscopy, flexible, transoral; with transendoscopic ultrasound-guided intramural or transmural fine needle aspiration/biopsy(s), esophagus (includes endoscopic ultrasound examination limited to the esophagus, stomach or duodenum, and adjacent structures) |
| 43239 | Esophagogastroduodenoscopy, flexible, transoral; with biopsy, single or multiple |
| 43240 | Esophagogastroduodenoscopy, flexible, transoral; with transmural drainage of pseudocyst (includes placement of transmural drainage catheter[s]/stent[s], when performed, and endoscopic ultrasound, when performed) |
| 43241 | Esophagogastroduodenoscopy, flexible, transoral; with insertion of intraluminal tube or catheter |
| 43242 | Esophagogastroduodenoscopy, flexible, transoral; with transendoscopic ultrasound-guided intramural or transmural fine needle aspiration/biopsy(s) (includes endoscopic ultrasound examination of the esophagus, stomach, and either the duodenum or a surgically altered stomach where the jejunum is examined distal to the anastomosis) |
| 43243 | Esophagogastroduodenoscopy, flexible, transoral; with injection sclerosis of esophageal/gastric varices |
| 43244 | Esophagogastroduodenoscopy, flexible, transoral; with band ligation of esophageal/gastric varices |
| 43245 | Esophagogastroduodenoscopy, flexible, transoral; with dilation of gastric/duodenal stricture(s) (eg, balloon, bougie) |
| 43246 | Esophagogastroduodenoscopy, flexible, transoral; with directed placement of percutaneous gastrostomy tube |
| 43247 | Esophagogastroduodenoscopy, flexible, transoral; with removal of foreign body |
| 43248 | Esophagogastroduodenoscopy, flexible, transoral; with insertion of guide wire followed by passage of dilator(s) through esophagus over guide wire |
| 43249 | Esophagogastroduodenoscopy, flexible, transoral; with transendoscopic balloon dilation of esophagus (less than 30 mm diameter) |
| 43250 | Esophagogastroduodenoscopy, flexible, transoral; with removal of tumor(s), polyp(s), or other lesion(s) by hot biopsy forceps or bipolar cautery |
| 43251 | Esophagogastroduodenoscopy, flexible, transoral; with removal of tumor(s), polyp(s), or other lesion(s) by snare technique |
| 43252 | Esophagogastroduodenoscopy, flexible, transoral; with optical endomicroscopy |
| 43255 | Esophagogastroduodenoscopy, flexible, transoral; with control of bleeding, any method |
| 43259 | Esophagogastroduodenoscopy, flexible, transoral; with endoscopic ultrasound examination, including the esophagus, stomach, and either the duodenum or a surgically altered stomach where the jejunum is examined distal to the anastomosis |
| 44360 | Small intestinal endoscopy, enteroscopy beyond second portion of duodenum, not including ileum; diagnostic, with or without collection of specimen(s) by brushing or washing (separate procedure) |
| 44361 | Small intestinal endoscopy, enteroscopy beyond second portion of duodenum, not including ileum; with biopsy, single or multiple |
| 44363 | Small intestinal endoscopy, enteroscopy beyond second portion of duodenum, not including ileum; with removal of foreign body |
| 44364 | Small intestinal endoscopy, enteroscopy beyond second portion of duodenum, not including ileum; with removal of tumor(s), polyp(s), or other lesion(s) by snare technique |
| 44365 | Small intestinal endoscopy, enteroscopy beyond second portion of duodenum, not including ileum; with removal of tumor(s), polyp(s), or other lesion(s) by hot biopsy forceps or bipolar cautery |
| 44366 | Small intestinal endoscopy, enteroscopy beyond second portion of duodenum, not including ileum; with control of bleeding (eg, injection, bipolar cautery, unipolar cautery, laser, heater probe, stapler, plasma coagulator) |
| 44369 | Small intestinal endoscopy, enteroscopy beyond second portion of duodenum, not including ileum; with ablation of tumor(s), polyp(s), or other lesion(s) not amenable to removal by hot biopsy forceps, bipolar cautery or snare technique |
| 44370 | Small intestinal endoscopy, enteroscopy beyond second portion of duodenum, not including ileum; with transendoscopic stent placement (includes predilation) |
| 44372 | Small intestinal endoscopy, enteroscopy beyond second portion of duodenum, not including ileum; with placement of percutaneous jejunostomy tube |
| 44373 | Small intestinal endoscopy, enteroscopy beyond second portion of duodenum, not including ileum; with conversion of percutaneous gastrostomy tube to percutaneous jejunostomy tube |
| 44376 | Small intestinal endoscopy, enteroscopy beyond second portion of duodenum, including ileum; diagnostic, with or without collection of specimen(s) by brushing or washing (separate procedure) |
| 44377 | Small intestinal endoscopy, enteroscopy beyond second portion of duodenum, including ileum; with biopsy, single or multiple |
| 44378 | Small intestinal endoscopy, enteroscopy beyond second portion of duodenum, including ileum; with control of bleeding (eg, injection, bipolar cautery, unipolar cautery, laser, heater probe, stapler, plasma coagulator) |
| 44379 | Small intestinal endoscopy, enteroscopy beyond second portion of duodenum, including ileum; with transendoscopic stent placement (includes predilation) |
| 44380 | Ileoscopy, through stoma; diagnostic, with or without collection of specimen(s) by brushing or washing (separate procedure) |
| 44382 | Ileoscopy, through stoma; with biopsy, single or multiple |
| 44383 | Ileoscopy, through stoma; with transendoscopic stent placement (includes predilation) |
| 44384 | Small bowel endoscopy |
| 44385 | Endoscopic evaluation of small intestinal (abdominal or pelvic) pouch; diagnostic, with or without collection of specimen(s) by brushing or washing (separate procedure) |
| 44386 | Endoscopic evaluation of small intestinal (abdominal or pelvic) pouch; with biopsy, single or multiple |
| 44388 | Colonoscopy through stoma; diagnostic, with or without collection of specimen(s) by brushing or washing (separate procedure) |
| 44389 | Colonoscopy through stoma; with biopsy, single or multiple |
| 44390 | Colonoscopy through stoma; with removal of foreign body |
| 44391 | Colonoscopy through stoma; with control of bleeding (eg, injection, bipolar cautery, unipolar cautery, laser, heater probe, stapler, plasma coagulator) |
| 44392 | Colonoscopy through stoma; with removal of tumor(s), polyp(s), or other lesion(s) by hot biopsy forceps or bipolar cautery |
| 44393 | Colonoscopy through stoma; with ablation of tumor(s), polyp(s), or other lesion(s) not amenable to removal by hot biopsy forceps, bipolar cautery or snare technique |
| 44394 | Colonoscopy through stoma; with removal of tumor(s), polyp(s), or other lesion(s) by snare technique |
| 44397 | Colonoscopy through stoma; with transendoscopic stent placement (includes predilation) |
| 45300 | Proctosigmoidoscopy, rigid; diagnostic, with or without collection of specimen(s) by brushing or washing (separate procedure) |
| 45305 | Proctosigmoidoscopy, rigid; with biopsy, single or multiple |
| 45307 | Proctosigmoidoscopy, rigid; with removal of foreign body |
| 45308 | Proctosigmoidoscopy, rigid; with removal of single tumor, polyp, or other lesion by hot biopsy forceps or bipolar cautery |
| 45309 | Proctosigmoidoscopy, rigid; with removal of single tumor, polyp, or other lesion by snare technique |
| 45315 | Proctosigmoidoscopy, rigid; with removal of multiple tumors, polyps, or other lesions by hot biopsy forceps, bipolar cautery or snare technique |
| 45317 | Proctosigmoidoscopy, rigid; with control of bleeding (eg, injection, bipolar cautery, unipolar cautery, laser, heater probe, stapler, plasma coagulator) |
| 45320 | Proctosigmoidoscopy, rigid; with ablation of tumor(s), polyp(s), or other lesion(s) not amenable to removal by hot biopsy forceps, bipolar cautery or snare technique (eg, laser) |
| 45321 | Proctosigmoidoscopy, rigid; with decompression of volvulus |
| 45327 | Proctosigmoidoscopy, rigid; with transendoscopic stent placement (includes predilation) |
| 45330 | Sigmoidoscopy, flexible; diagnostic, with or without collection of specimen(s) by brushing or washing (separate procedure) |
| 45331 | Sigmoidoscopy, flexible; with biopsy, single or multiple |
| 45332 | Sigmoidoscopy, flexible; with removal of foreign body |
| 45333 | Sigmoidoscopy, flexible; with removal of tumor(s), polyp(s), or other lesion(s) by hot biopsy forceps or bipolar cautery |
| 45334 | Sigmoidoscopy, flexible; with control of bleeding (eg, injection, bipolar cautery, unipolar cautery, laser, heater probe, stapler, plasma coagulator) |
| 45335 | Sigmoidoscopy, flexible; with directed submucosal injection(s), any substance |
| 45337 | Sigmoidoscopy, flexible; with decompression of volvulus, any method |
| 45338 | Sigmoidoscopy, flexible; with removal of tumor(s), polyp(s), or other lesion(s) by snare technique |
| 45339 | Sigmoidoscopy, flexible; with ablation of tumor(s), polyp(s), or other lesion(s) not amenable to removal by hot biopsy forceps, bipolar cautery or snare technique |
| 45340 | Sigmoidoscopy, flexible; with dilation by balloon, 1 or more strictures |
| 45341 | Sigmoidoscopy, flexible; with endoscopic ultrasound examination |
| 45342 | Sigmoidoscopy, flexible; with transendoscopic ultrasound guided intramural or transmural fine needle aspiration/biopsy(s) |
| 45345 | Sigmoidoscopy, flexible; with transendoscopic stent placement (includes predilation) |
| 45378 | Colonoscopy, flexible, proximal to splenic flexure; diagnostic, with or without collection of specimen(s) by brushing or washing, with or without colon decompression (separate procedure) |
| 45379 | Colonoscopy, flexible, proximal to splenic flexure; with removal of foreign body |
| 45380 | Colonoscopy, flexible, proximal to splenic flexure; with biopsy, single or multiple |
| 45381 | Colonoscopy, flexible, proximal to splenic flexure; with directed submucosal injection(s), any substance |
| 45382 | Colonoscopy, flexible, proximal to splenic flexure; with control of bleeding (eg, injection, bipolar cautery, unipolar cautery, laser, heater probe, stapler, plasma coagulator) |
| 45383 | Colonoscopy, flexible, proximal to splenic flexure; with ablation of tumor(s), polyp(s), or other lesion(s) not amenable to removal by hot biopsy forceps, bipolar cautery or snare technique |
| 45384 | Colonoscopy, flexible, proximal to splenic flexure; with removal of tumor(s), polyp(s), or other lesion(s) by hot biopsy forceps or bipolar cautery |
| 45385 | Colonoscopy, flexible, proximal to splenic flexure; with removal of tumor(s), polyp(s), or other lesion(s) by snare technique |
| 45386 | Colonoscopy, flexible, proximal to splenic flexure; with dilation by balloon, 1 or more strictures |
| 45387 | Colonoscopy, flexible, proximal to splenic flexure; with transendoscopic stent placement (includes predilation) |
| 45391 | Colonoscopy, flexible, proximal to splenic flexure; with endoscopic ultrasound examination |
| 45392 | Colonoscopy, flexible, proximal to splenic flexure; with transendoscopic ultrasound guided intramural or transmural fine needle aspiration/biopsy(s) |
| 46600 | Anoscopy; diagnostic, with or without collection of specimen(s) by brushing or washing (separate procedure) |
| 46604 | Anoscopy; with dilation (eg, balloon, guide wire, bougie) |
| 46606 | Anoscopy; with biopsy, single or multiple |
| 46608 | Anoscopy; with removal of foreign body |
| 46610 | Anoscopy; with removal of single tumor, polyp, or other lesion by hot biopsy forceps or bipolar cautery |
| 46611 | Anoscopy; with removal of single tumor, polyp, or other lesion by snare technique |
