# Supplementary material for: Capnography sensor use is associated with reduction of adverse outcomes during gastrointestinal endoscopic procedures with sedation administration
Source: BMC Anesthesiol. 2017 Nov 28;17:157. doi: 10.1186/s12871-017-0453-9 (PMC5704394; doi:10.1186/s12871-017-0453-9)
Supplement: Supplementary file 2 — Capnography Sensor Codes. (DOCX 178 kb) [file 12871_2017_453_MOESM2_ESM.docx]

**Supplemental Table 2. Capnography Sensor Codes**

| **Hospital Charge Description** | **Hospital Charge Description** |
| --- | --- |
| 272-CO2 DETECTOR END TIDAL DISP | DETECTOR,CO2 |
| ADULT CO2 DETECTOR | END CO2 NASAL CANNULA W O2 |
| CANN ETCO2 CAPNO ADULT 13FT | END TIDAL CO2 |
| CANN ETCO2 CAPNO W/BITE BLOCK | END TIDAL CO2 DAILY |
| CANN NSL L7FT CO2 ADLT | END TIDAL CO2 DETECTOR |
| CANNUAL CO2 NASAL | END TIDAL CO2 DEVICE |
| CANNULA 02/CO2 MICROSTREAM | END TIDAL CO2 MODULE |
| CANNULA CAPNOGRAPHY | END TIDAL CO2 MONITOR SET |
| CANNULA CO2 | END TIDAL CO2 MONITOR SET UP |
| CANNULA CO2 NASAL | END TIDAL CO2 MONITOR, RE |
| CANNULA CO2 NASAL | END TIDAL CO2 MONT/DAY |
| CANNULA CO2 NASAL SAMPLING | END TIDAL CO2 PER HR |
| CANNULA NASAL CO2 | END TIDAL CO2 PT/SYSTEM ASSESS |
| CANNULA NASAL CO2 | END TIDAL CO2 SET UP COUNT |
| CANNULA NASAL CO2 MONITORING | END TIDAL CO2 THER N/C |
| CANNULA NASAL CO2 W/O2 | END-TIDAL CO2 DETECTOR |
| CANNULA NASAL ETCO2 | ENDTIDAL CO2 INITIAL SUPPLY |
| CANNULA O2 NASAL W/CO2 CONNECT | ET TUBE CO2 DETECTOR |
| CANNULA O2/CO2 (0-50) | ETCO2 CANNULA |
| CANNULA ORAL NASAL/ET CO2 | ETCO2 CHECK |
| CANNULA ORAL/NASAL CO2 SAMPLE | ETCO2 DETECTOR DISPOSABLE |
| CANNULA OW/CO2 ORAL NASAL DISP | ETCO2 DETECTORS |
| CANNULA, O2 W/CO2 MONITORING | ETCO2 FILTER LINE |
| CANNULA, O2 W/CO2 MONITORING | ETCO2 SENSOR |
| CANNULA, O2 W/CO2 MONITORING PEDS | ETCO2 SUPPLIES |
| CANNULA,CO2 SAMPLING PEDS | ETCO2 TUBING FILTER |
| CANNULA,NASAL CO2 FOR PEDS | EXHALED CO2 MONITORING |
| CANNULANASALETCO2 | FILTER CO2 PN10FILTPK02 |
| CANNULAS 008177 ADULT TITAL CO2 | FILTERLINE CO2 SET |
| CAPNOGRAPHY CANNULA | FILTERLINE ETCO2 ADULT/PEDS |
| CAPNOGRAPHY SAMPLE TUBING | FILTERLINE ETCO2 SET ADULT/PDS |
| CAPNOLINE H CO2 INF/NEO | Filterline H Set ET/CO2 AD/PED |
| CIRCUIT FILTERLINE H PED ETCO2 | FLOW SENSOR |
| CIRCUIT MICROSTREAM ETCO2 | FLOW SENSOR CO2 |
| CIRCUITFILTERLNEHADLTETCO2 | FLOW SENSOR RESP SUPPLY CAT#53 |
| CO2 AIRWAY ADAPTER SENSOR | HC CO2 CANNULA |
| CO2 CAD DETECTOR | HC CO2 CANNULA |
| CO2 CANNULA | HC END TIDAL CO2 |
| CO2 DETECTOR | HC ET CO2 SET UP SUPPLIES |
| CO2 DETECTOR | ICU CO2 MONITORING |
| CO2 DETECTOR | INFANT FLOWSENSOR |
| CO2 DETECTOR (ADULT) DISP | KIT ETCO2 |
| CO2 DETECTOR (INTUBATION) | KIT,ETCO2 SAMPLING 28757 |
| CO2 DETECTOR ADULT | LINE ANES CO2 SAMPLING 10 FT |
| CO2 Detector Adult 562134 | MAC NASAL CANNULA W/ CO2 LINE |
| CO2 Detector Disposable | MONITOR END TIDAL CO2 PER HR |
| CO2 DETECTOR END TIDAL | NASAL CAN/CO2 LINE |
| CO2 DETECTOR END TIDAL DISP | NASAL CANN W CO2 SAMP LNE |
| CO2 DETECTOR PEDI | NASAL CANN W CO2 SMP LNE |
| CO2 DETECTOR PEDIATRIC | NASAL CANNULA W CO2 LINE |
| CO2 DETECTORS | NASAL CANNULA WITH CO2 ADULT |
| CO2 END TIDAL MONITOR DISP | NASAL CANNULA WITH CO2 PEDS |
| CO2 FILTER | NASAL CO2 SAMPLING CANNULA-ADULT |
| CO2 FILTER LINE O2 NC | NEONATAL ETCO2 CANNULA |
| CO2 FILTERLINE 02 NC | NEONATAL ETCO2 CANNULA |
| CO2 FILTERLINE H | NONIVA MEASURE ETCO2 |
| CO2 FLOW SENSOR ADULT | NP END TIDAL CO2 INITIAL/SHIFT |
| CO2 KIT | O2 CANNUAL W/7CO2 SAMPLE LINE |
| CO2 MONITOR | O2 CO2 NASAL CANULA |
| CO2 MONITOR INITIAL DAY | O2/CO2 NASAL FILTERLINE A |
| CO2 MONITORING LINE-COMBO | O2/CO2 SUPPLIES |
| CO2 NASAL CANNULA W/O2 | PED ETCO2 DAILY |
| CO2 NASAL CANULA | PED ETCO2 SUPPLIES |
| CO2 ORAL/NASAL CANNULA | PEDI CO2 DETECTOR |
| CO2 PT FILTER LINE | PEDI CO2 DETECTOR |
| CO2 SENSOR | PEDIATRIC CO2 DETECTOR |
| CO2 TUBING | PEDICAP CO2 DETECTOR |
| CO2 TUBING DISP | RC ENDTIDAL CO2 SUPPLY |
| CP END TIDAL CO2 SUPPLY | SENSOR CAPOLINE PS 02 ADULT |
| DETECT CO2 DISP PEDI | SENSOR CAPOLINE PS 02 PEDS |
| DETECTOR CO2 | SENSOR FLOW ADULT CO2 |
| DETECTOR CO2 ADLT 25CC ET TB P | SENSOR NICO ADULT CO2 FLOW |
| DETECTOR CO2 ADLT 25CC ET TB P | SENSOR NICO ADULT CO2 FLOW |
| DETECTOR CO2 ADULT | SENSOR, ADULT MASIMO SPO2 DISP |
| Detector CO2 Adult 562134-10 | SENSOR, CO2 MONITORI |
| DETECTOR CO2 DIS | SENSOR, ETCO2 |
| DETECTOR CO2 END TIDAL | SET CO2 |
| DETECTOR CO2 END TIDAL PEDS | SET FILTERLINE CO2 |
| DETECTOR CO2 PEDIATRIC | TC CO2 MONITER SETUP |
| DETECTOR END TIDAL CO2 | TCOM/ETCO2 MONITOR |
| DETECTOR END TIDAL CO2 24/CS | TCOM/ETCO2 MONITOR SET UP |
| DETECTOR END TIDAL CO2 D | TUBING CO2 620-040-660 |
| DETECTOR END TIDALCO2 PED | TUBING ETCO2 FOR MICROSTRM |
| DETECTOR END-TIDAL CO2 | TUBING WITH FILTER CO2 |
| DETECTOR ETCO2 | TUBING, MICROSTREAM O2/CO2 NASAL FILTER |
