# Supplementary material for: Capnography sensor use is associated with reduction of adverse outcomes during gastrointestinal endoscopic procedures with sedation administration
Source: BMC Anesthesiol. 2017 Nov 28;17:157. doi: 10.1186/s12871-017-0453-9 (PMC5704394; doi:10.1186/s12871-017-0453-9)
Supplement: Supplementary file 3 — Variables Included in the Multivariable Logistic Regression. (DOCX 67 kb) [file 12871_2017_453_MOESM3_ESM.docx]

**Supplemental Table 3.**

| **Variables Included in the Multivariable Logistic Regression** |
| --- |
| - Patient Characteristics:   - Age   - Gender   - Race - Hospital Characteristics:   - Region   - Teaching Status   - Bed Size   - Rural vs. Urban - Charlson Comorbidities:   - Myocardial infarction   - Congestive heart failure   - Dementia   - Chronic obstructive pulmonary disease (COPD)   - Rheumatoid arthritis   - Peptic ulcer disease   - Paralysis   - Chronic renal failure   - Cancer   - Metastatic solid tumor   - Acquired immunodeficiency syndrome (AIDS)   - Obesity   - Diabetes   - Hypertension   - Peripheral vascular disease   - Cardiovascular disease   - Mild liver disease   - Moderate-severe liver disease |
