# Supplementary material for: Capnography sensor use is associated with reduction of adverse outcomes during gastrointestinal endoscopic procedures with sedation administration
Source: BMC Anesthesiol. 2017 Nov 28;17:157. doi: 10.1186/s12871-017-0453-9 (PMC5704394; doi:10.1186/s12871-017-0453-9)
Supplement: Supplementary file 4 — Propensity Score Matching – Inpatient Population. (DOCX 170 kb) [file 12871_2017_453_MOESM4_ESM.docx]

**Supplemental Table 4. PS Matching – Inpatient Population**

| **Characteristics** | **Before Match** | | | **After Match** | | |
| --- | --- | --- | --- | --- | --- | --- |
|  | **Capnography ± SpO_2_ (n = 5,146)** | **SpO_2_ Only**  **(n = 19,308)** | **Standard Difference** | **Capnography ± SpO_2_ (n = 4,771)** | **SpO_2_ Only**  **(n = 4,771)** | **Standard Difference** |
| **Age (mean)** | 64.27 | 63.43 | 0.047 | 64.12 | 63.90 | 0.012 |
| **CCI (mean)** | 2.53 | 2.59 | -0.021 | 2.55 | 2.51 | 0.019 |
| **Male** | 48.04% | 48.34% | -0.006 | 47.66% | 49.49% | -0.036 |
| **Race** |  |  |  |  |  |  |
| White | 66.40% | 72.67% | **-0.137** | 65.44% | 66.51% | -0.023 |
| Black | 17.64% | 14.27% | 0.092 | 18.05% | 15.64% | 0.064 |
| Hispanic | 3.19% | 1.99% | 0.075 | 3.21% | 3.31% | -0.006 |
| Other | 12.77% | 11.06% | 0.053 | 13.31% | 14.55% | -0.036 |
| **APR severity of illness** |  |  |  |  |  |  |
| 1=mild | 10.30% | 9.07% | 0.042 | 10.21% | 10.21% | 0.000 |
| 2=moderate | 33.66% | 29.11% | 0.098 | 32.53% | 32.78% | -0.005 |
| 3=severe | 42.29% | 42.90% | -0.012 | 42.65% | 41.48% | 0.024 |
| 4=extreme | 13.76% | 18.92% | **-0.140** | 14.61% | 15.53% | -0.026 |
| **Comorbidity** |  |  |  |  |  |  |
| HTN | 64.69% | 62.56% | 0.044 | 64.83% | 63.36% | 0.031 |
| Diabetes | 31.64% | 30.71% | 0.020 | 31.78% | 30.62% | 0.025 |
| COPD | 24.10% | 24.48% | -0.009 | 24.25% | 24.00% | 0.006 |
| CRF | 20.52% | 21.08% | -0.014 | 20.75% | 20.50% | 0.006 |
| CHF | 16.11% | 19.10% | -0.079 | 16.20% | 16.77% | -0.015 |
| PUD | 13.12% | 14.23% | -0.032 | 13.37% | 13.92% | -0.016 |
| Obesity | 10.90% | 11.01% | -0.003 | 11.09% | 10.98% | 0.003 |
| Cancer | 10.40% | 9.82% | 0.019 | 10.42% | 10.17% | 0.008 |
| MLD | 10.01% | 10.42% | -0.013 | 10.23% | 10.19% | 0.001 |
| MSLD | 9.52% | 10.41% | -0.029 | 9.77% | 9.87% | -0.004 |
| MI | 8.16% | 8.87% | -0.025 | 8.30% | 8.38% | -0.003 |
| PVD | 6.69% | 7.28% | -0.023 | 6.77% | 6.96% | -0.007 |
| CVD | 6.02% | 6.75% | -0.030 | 6.06% | 6.25% | -0.008 |
| MST | 5.21% | 4.74% | 0.021 | 5.20% | 4.63% | 0.026 |
| RA | 3.11% | 3.40% | -0.017 | 3.16% | 3.08% | 0.005 |
| Dementia | 0.70% | 0.89% | -0.022 | 0.75% | 0.80% | -0.005 |
| Paralysis | 0.70% | 0.89% | -0.022 | 0.75% | 0.67% | 0.010 |
| AIDS | 0.49% | 0.34% | 0.023 | 0.44% | 0.42% | 0.003 |
| **Region** |  |  |  |  |  |  |
| South | 63.78% | 74.77% | **-0.240** | 68.77% | 68.12% | 0.014 |
| Northeast | 2.33% | 7.99% | **-0.258** | 2.52% | 2.22% | 0.019 |
| Midwest | 18.87% | 6.79% | **0.367** | 12.89% | 15.18% | -0.066 |
| West | 15.02% | 10.45% | **0.137** | 15.83% | 14.48% | 0.037 |
| **Teaching hospital** | 18.15% | 44.91% | **-0.601** | 19.56% | 20.35% | -0.020 |
| **Hospital Bed size** |  |  |  |  |  |  |
| < 250 | 17.55% | 13.07% | **0.125** | 18.53% | 19.51% | -0.025 |
| 250 - 500 | 54.96% | 40.15% | **0.300** | 51.86% | 50.51% | 0.027 |
| 500 + | 27.50% | 46.78% | **-0.407** | 29.62% | 29.97% | -0.008 |
| **Hospital Location** |  |  |  |  |  |  |
| Urban | 77.24% | 87.98% | **-0.286** | 81.97% | 82.23% | -0.007 |

Values presented as mean, %, and standard difference. Standard differences > 0.10 are in bold font. AIDS = acquired immune deficiency virus; APR = all patient refined; CCI = Charlson comorbidity index; CHF = congestive heart failure; COPD = chronic obstructive pulmonary disease; CRF = chronic renal failure; CVD = cardiovascular disease; HTN = hypertension; MI = myocardial infarction; MLD = mild liver disease; MSLD = moderate-severe liver disease; MST = metastatic solid tumor; PUD = peptic ulcer disease; PVD = peripheral vascular disease; RA = rheumatoid arthritis.
