# Supplementary material for: Capnography sensor use is associated with reduction of adverse outcomes during gastrointestinal endoscopic procedures with sedation administration
Source: BMC Anesthesiol. 2017 Nov 28;17:157. doi: 10.1186/s12871-017-0453-9 (PMC5704394; doi:10.1186/s12871-017-0453-9)
Supplement: Supplementary file 5 — Propensity Score Matching – Outpatient Population. (DOCX 155 kb) [file 12871_2017_453_MOESM5_ESM.docx]

**Supplemental Table 5. PS Matching – Outpatient Population**

| **Characteristics** | **Before Match** | | | **After Match** | | |
| --- | --- | --- | --- | --- | --- | --- |
|  | **Capnography ± SpO_2_ (n = 62,315)** | **SpO_2_ Only**  **(n = 131,292)** | **Standard Difference** | **Capnography ± SpO_2_ (n = 35,130)** | **SpO_2_ Only**  **(n = 35,130)** | **Standard Difference** |
| **Age (mean)** | 58.33 | 53.36 | **0.275** | 56.70 | 57.81 | -0.060 |
| **CCI (mean)** | 0.47 | 0.51 | -0.038 | 0.58 | 0.62 | -0.031 |
| **Male** | 43.03% | 43.08% | -0.001 | 42.43% | 42.37% | 0.001 |
| **Race** |  |  |  |  |  |  |
| White | 77.02% | 76.96% | 0.002 | 73.40% | 77.45% | -0.094 |
| Black | 10.69% | 9.00% | 0.057 | 10.01% | 10.11% | -0.004 |
| Hispanic | 1.98% | 3.04% | -0.068 | 3.39% | 2.69% | 0.041 |
| Other | 10.30% | 11.01% | -0.023 | 13.21% | 9.75% | **0.109** |
| **Comorbidity** |  |  |  |  |  |  |
| HTN | 32.48% | 30.65% | 0.039 | 35.19% | 34.79% | 0.008 |
| Diabetes | 13.50% | 13.33% | 0.005 | 15.27% | 15.44% | -0.005 |
| COPD | 8.17% | 8.88% | **-0.252** | 10.39% | 11.56% | -0.037 |
| PUD | 3.54% | 4.15% | -0.032 | 4.45% | 4.82% | -0.018 |
| Obesity | 2.87% | 4.59% | -0.091 | 4.45% | 5.29% | -0.039 |
| Cancer | 2.72% | 3.02% | -0.018 | 3.61% | 3.52% | 0.005 |
| MI | 1.76% | 1.65% | 0.008 | 2.09% | 2.11% | -0.001 |
| MSLD | 1.58% | 1.67% | -0.007 | 1.83% | 2.12% | -0.020 |
| MLD | 1.40% | 1.46% | -0.005 | 1.63% | 1.79% | -0.013 |
| CHF | 1.25% | 1.74% | -0.040 | 1.97% | 2.08% | -0.008 |
| RA | 1.21% | 1.11% | 0.010 | 1.31% | 1.32% | 0.000 |
| CRF | 1.05% | 1.26% | -0.020 | 1.51% | 1.73% | -0.018 |
| PVD | 0.64% | 0.82% | **-0.219** | 0.95% | 1.02% | -0.007 |
| CVD | 0.35% | 0.58% | **-0.338** | 0.58% | 0.72% | -0.017 |
| MST | 0.33% | 0.38% | -0.009 | 0.47% | 0.50% | -0.004 |
| AIDS | 0.05% | 0.05% | -0.004 | 0.06% | 0.05% | 0.007 |
| Paralysis | 0.05% | 0.07% | -0.009 | 0.08% | 0.08% | -0.001 |
| Dementia | 0.03% | 0.03% | 0.002 | 0.05% | 0.04% | 0.007 |
| **Region** |  |  |  |  |  |  |
| South | 65.75% | 81.60% | **-0.366** | 79.78% | 82.66% | -0.074 |
| Northeast | 11.94% | 1.85% | **0.406** | 0.73% | 1.42% | -0.067 |
| Midwest | 12.36% | 6.92% | **0.185** | 3.50% | 2.97% | 0.030 |
| West | 9.95% | 9.62% | 0.011 | 16.00% | 12.95% | 0.087 |
| **Teaching hospital** | 16.29% | 31.31% | **-0.358** | 24.56% | 25.72% | -0.027 |
| **Hospital Bed size** |  |  |  |  |  |  |
| < 250 | 31.62% | 29.23% | 0.052 | 34.18% | 37.23% | -0.064 |
| 250 - 500 | 55.70% | 19.50% | **0.806** | 43.33% | 41.00% | 0.047 |
| 500 + | 12.68% | 51.27% | **-0.909** | 22.49% | 21.77% | 0.017 |
| **Hospital Location** |  |  |  |  |  |  |
| Urban | 85.62% | 79.35% | **0.166** | 85.96% | 87.44% | -0.044 |

Values presented as mean, %, and standard difference. Standard differences > 0.10 are in bold font. AIDS = acquired immune deficiency virus; APR = all patient refined; CCI = Charlson comorbidity index; CHF = congestive heart failure; COPD = chronic obstructive pulmonary disease; CRF = chronic renal failure; CVD = cardiovascular disease; HTN = hypertension; MI = myocardial infarction; MLD = mild liver disease; MSLD = moderate-severe liver disease; MST = metastatic solid tumor; PUD = peptic ulcer disease; PVD = peripheral vascular disease; RA = rheumatoid arthritis.
