# Supplementary material for: Capnography sensor use is associated with reduction of adverse outcomes during gastrointestinal endoscopic procedures with sedation administration
Source: BMC Anesthesiol. 2017 Nov 28;17:157. doi: 10.1186/s12871-017-0453-9 (PMC5704394; doi:10.1186/s12871-017-0453-9)
Supplement: Supplementary file 6 — Patient Outcomes Before and After Propensity Score Matching – Inpatient Population. (DOCX 63 kb) [file 12871_2017_453_MOESM6_ESM.docx]

**Supplemental Table 6. Patient Outcomes Before and After PS Matching – Inpatient Population**

| **Outcome** | **Before Match** | | | **After Match** | | |
| --- | --- | --- | --- | --- | --- | --- |
|  | **Capnography ± SpO_2_ (n = 5,146)** | **SpO_2_ Only**  **(n = 19,308)** | **P-value** | **Capnography ± SpO_2_ (n = 4,771)** | **SpO_2_ Only**  **(n = 4,771)** | **P-value** |
| Death | 100 (1.94%) | 792 (4.10%) | < 0.0001 | 94 (1.97%) | 166 (3.48%) | < 0.0001 |
| Rescue Event | 69 (1.34%) | 325 (1.68%) | 0.083 | 66 (1.38%) | 74 (1.55%) | 0.5 |
