# Supplementary material for: Capnography sensor use is associated with reduction of adverse outcomes during gastrointestinal endoscopic procedures with sedation administration
Source: BMC Anesthesiol. 2017 Nov 28;17:157. doi: 10.1186/s12871-017-0453-9 (PMC5704394; doi:10.1186/s12871-017-0453-9)
Supplement: Supplementary file 7 — Patient Outcomes Before and After Propensity Score Matching – Outpatient Population. (DOCX 63 kb) [file 12871_2017_453_MOESM7_ESM.docx]

**Supplemental Table 7. Patient Outcomes Before and After PS Matching – Outpatient Population**

| **Outcome** | **Before Match** | | | **After Match** | | |
| --- | --- | --- | --- | --- | --- | --- |
|  | **Capnography ± SpO_2_ (n = 62,315)** | **SpO_2_ Only**  **(n = 131,292)** | **P-value** | **Capnography ± SpO_2_ (n = 35,130)** | **SpO_2_ Only**  **(n = 35,130)** | **P-value** |
| Death | 1 (< 0.01%) | 10 (0.01%) | 0.12 | 1 (< 0.01%) | 4 (0.01%) | 0.38 |
| Rescue Event | 129 (0.21%) | 466 (0.35%) | < 0.0001 | 63 (0.18%) | 148 (0.42%) | < 0.0001 |
